# Mammographic density and exposure to air pollutants in premenopausal women: a cross-sectional study

**DOI:** 10.1265/ehpm.24-00209

**Published:** 2024-11-22

**Authors:** Tamara Jiménez, Alejandro Domínguez-Castillo, Nerea Fernández de Larrea-Baz, Pilar Lucas, María Ángeles Sierra, Sergio Maeso, Rafael Llobet, Marina Nieves Pino, Mercedes Martínez-Cortés, Beatriz Pérez-Gómez, Marina Pollán, Virginia Lope, Javier García-Pérez

**Affiliations:** 1 Department of Preventive Medicine, Public Health and Microbiology, Universidad Autónoma de Madrid (UAM), C/Arzobispo Morcillo, 4, 28029, Madrid, Spain; 2 HM CINAC (Centro Integral de Neurociencias AC), Hospital Universitario Puerta del Sur, Fundación HM Hospitales, Av. Carlos V, 70, 28938 Móstoles, Spain; 3 Cancer and Environmental Epidemiology Unit, Department of Epidemiology of Chronic Diseases, National Center for Epidemiology, Carlos III Institute of Health (Instituto de Salud Carlos III), Avda. Monforte de Lemos, 5, 28029 Madrid, Spain; 4 Consortium for Biomedical Research in Epidemiology & Public Health (CIBER en Epidemiología y Salud Pública – CIBERESP), Avda. Monforte de Lemos, 3-5, 28029 Madrid, Spain; 5 Institute of Computer Technology, Universitat Politècnica de València, Valencia, Spain; 6 Servicio de Prevención y Promoción de la Salud, Madrid Salud, Madrid City Council, 62 Mediterraneo Avenue, Floor 6, Madrid, Spain; 7 Madrid Salud, Madrid City Council, 62 Mediterraneo Avenue, Floor 6, Madrid, Spain

**Keywords:** Breast density, Air pollution, Kriging, Correlation, Principal component analysis, Breast cancer, DDM-Madrid, Long-term exposure

## Abstract

**Background:**

Mammographic density (MD) is a well-established risk factor for breast cancer. Air pollution is a major public health concern and a recognized carcinogen. We aim to investigate the association between MD and exposure to specific air pollutants (SO_2_, CO, NO, NO_2_, NO_x_, PM_2.5_, PM_10_, and O_3_) in premenopausal females.

**Methods:**

This cross-sectional study, carried out in Spain, included 769 participants who attended their gynecological examinations. Hourly concentrations of the pollutants were extracted from the Air Quality Monitoring System of Madrid City over a 3-year period. Individual long-term exposure to pollutants was assessed by geocoding residential addresses and monitoring stations, and applying ordinary kriging to the 3-year annual mean concentrations of each pollutant to interpolate the surface of Madrid. This exposure variable was categorized into quartiles. In a first analysis, we used multiple linear regression models with the log-transformed percent MD as a continuous variable. In a second analysis, we used MD as a dichotomous variable (“high” density (MD > 50%) *vs.* “low” density (MD ≤ 50%)) and applied multiple logistic regression models to estimate odds ratios (ORs). We also analyzed the correlation among the pollutants, and performed a principal component analysis (PCA) to reduce the dimensionality of this set of eight correlated pollutants into a smaller set of uncorrelated variables (principal components (PCs)). Finally, the initial analyses were applied to the PCs to detect underlying patterns of emission sources.

**Results:**

The first analysis detected no association between MD and exposure to any of the pollutants. The second analysis showed non-statistically significant increased risks (OR_Q4_; IC95%) of high MD were detected in women with higher exposure to SO_2_ (1.50; 0.90–2.48), and PM_2.5_ (1.27; 0.77–2.10). In contrast, non-significant ORs < 1 were found in all exposure quartiles for NO (OR_Q2_ = 0.72, OR_Q3_ = 0.68, OR_Q4_ = 0.78), and PM_10_ (OR_Q2_ = 0.69, OR_Q3_ = 0.82, OR_Q4_ = 0.72). PCA identified two PCs (PC1: “traffic pollution” and PC2: “natural pollution”), and no association was detected between MD and proximity to these two PCs.

**Conclusions:**

In general, our results show a lack of association between residential exposure to specific air pollutants and MD in premenopausal females. Future research is needed to confirm or refute these findings.

## Graphical Abstract

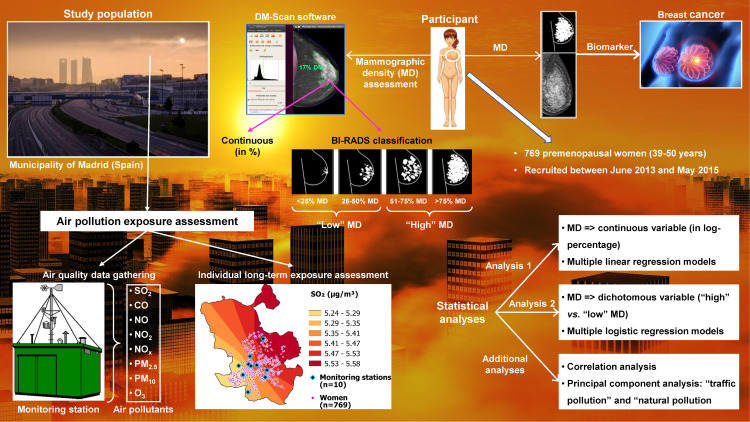

## 1. Background

Breast cancer is the leading cause of female cancer worldwide [1] and represents a major health problem, as its incidence is expected to rise in the next decade in our country [2]. Regarding environmental exposures, ambient air pollution was classified as carcinogenic to humans [3] because it contains various carcinogens and endocrine-disrupting chemicals (EDCs). In urban zones, traffic-related air pollution (TRAP) is a significant contributor, which includes substances such as nitrogen oxides (NO_x_) and particulate matter (PM) [4].

Some authors have attempted to establish a link between ambient pollutants in urban areas and increased female gynecological cancers [5], and more specifically with breast cancer risk, although with inconclusive findings [6–8]. Nevertheless, some studies have found that women living in environments with high levels of air pollution have an increased risk of developing breast cancer. This increased risk has been associated with exposure to specific pollutants, such as nitrogen dioxide (NO_2_) [9, 10], PM [11], and sulfur dioxide (SO_2_) [12]. In the case of SO_2_, it has been proposed that its ability to absorb ultraviolet light could interfere with vitamin D synthesis in the skin, leading to vitamin D deficiency and, consequently, an increased risk of breast cancer [13]. In addition, exposure to air pollutants has been shown to be associated with an increased frequency of genetic damage, including changes in DNA methylation patterns and telomere shortening [14–17]. These alterations may influence the expression of genes related to DNA repair, cell cycle control, inflammation and the response to oxidative stress [18]. In particular, exposure to PM, which is composed of a variety of substances with endocrine and carcinogenic properties [19], has been linked to accelerated biological age, as measured by DNA methylation [20].

Mammographic density (MD), the proportion of radiologically dense fibro-glandular tissue in the breast [21], is a well-established risk factor for breast cancer. An important characteristic of this biomarker is its dynamic and modifiable nature, which can be altered by various factors, including parity, body mass index (BMI), age, and menopausal transition [22]. The importance of this biomarker for breast cancer risk is such that it has been established that the risk of breast cancer can be up to 6 times higher in women with MD above 75% compared to those with lower densities (MD < 5%) [21, 23]. Regarding MD and air pollution exposure, the available literature focusing on specific pollutants (PM, NO_x_, or ozone (O_3_)) is scarce, and shows inconsistent findings [24–30]. Investigating the possible effect of certain air pollutants on MD may help to clarify the causal mechanism of the associations detected with breast cancer found in previous studies. It is also the risk factor with the highest attributable fraction [31], and its inclusion in the breast cancer risk prediction models improves the estimate [32]. Therefore, we aimed to assess whether there is a relationship between MD and exposure to eight air pollutants (SO_2_, carbon monoxide (CO), nitrogen monoxide (NO), NO_2_, NO_x_, PM < 2.5 µm (PM_2.5_), PM < 10 µm (PM_10_), and O_3_) in premenopausal Spanish women.

## 2. Methods

### 2.1. Study population

**Fig. 1:**
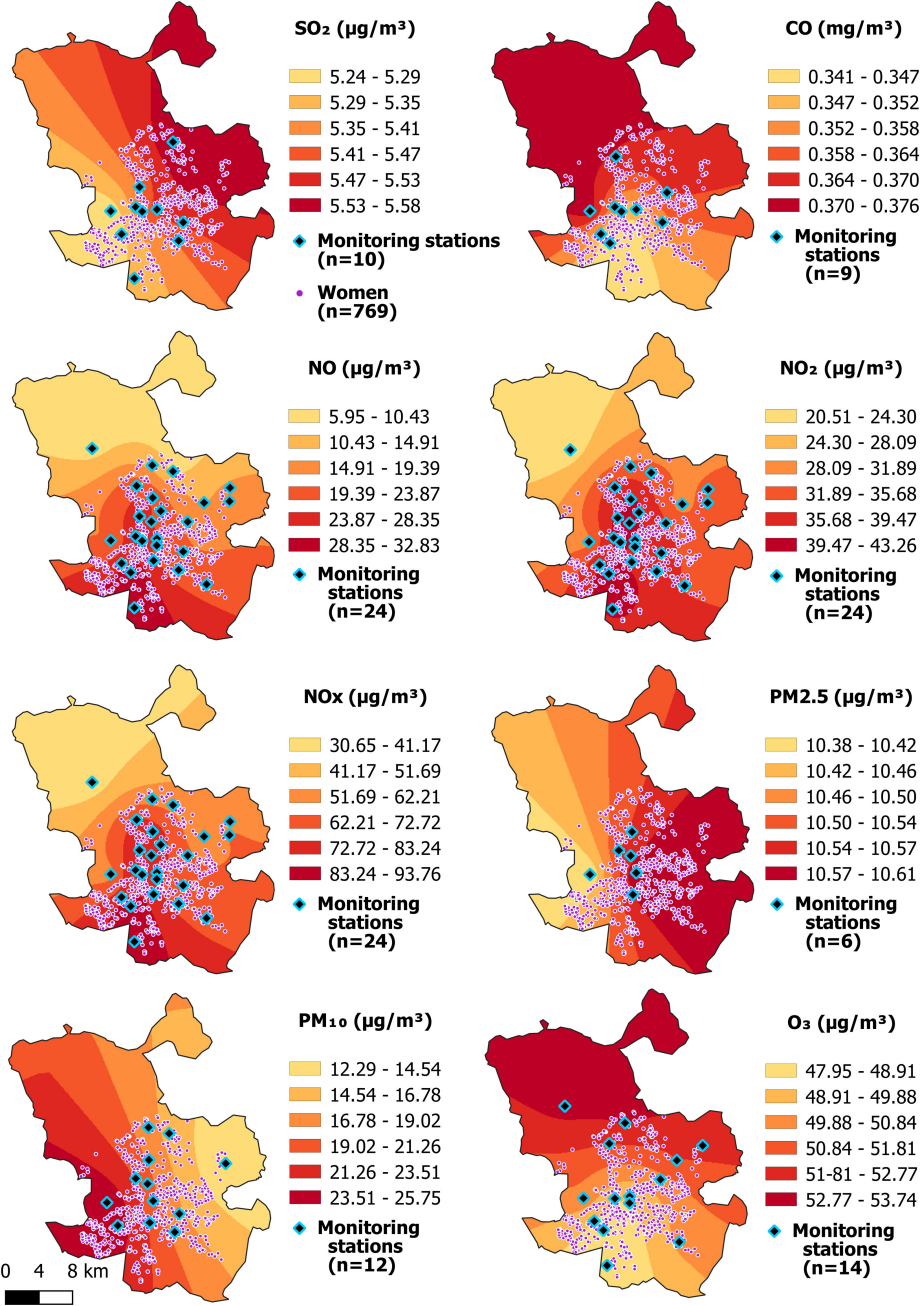
Concentrations of pollutants interpolated by kriging, and locations of women’s addresses and monitoring stations.

DDM-Madrid is a Spanish cross-sectional study carried out from 2013 to 2015 [33]. A total of 1466 premenopausal women (39–50 years) were recruited from the Madrid Medical Diagnostic Center during their routine gynecological examinations. Participants were contacted by phone, and 88% of them agreed to participate in the study providing their written informed consents. Trained personnel administered an epidemiological questionnaire on the same day as participants’ medical examinations. The comprehensive data collection covered sociodemographic information, medical history, lifestyle, and residential history. Geographical data were obtained by geocoding each participant’s address into EPSG:23030 coordinates. We excluded 24 participants with missing data on the address variable, and restricted the analysis to the 896 women residing in the municipality of Madrid. Madrid is the second most populated city in the EU (3.3 million inhabitants), and it experiences intense road traffic, with high NO_2_ levels exceeding EU limit values [34]. After excluding 11 women whose MD could not be measured, 18 participants with analogical images, and 98 participants with missing data in covariates, the final sample comprised 769 women, whose geographic distribution is depicted in Fig. 1.

### 2.2. Mammographic density assessment

The 2D digital mammograms of both breasts were collected in craniocaudal and mediolateral oblique views. An experienced radiologist assessed the % of MD from the craniocaudal view of the left breast using DM-Scan, a semi-automated computer tool with high validity and reproducibility [35]. Additionally, we employed the American College of Radiology’s Breast Imaging-Reporting and Data System (BI-RADS) classification [36], to categorize breast density based on the amount of fibroglandular tissue present. The classification includes: category 1) almost entirely fat (<25% of fibroglandular tissue); category 2) scattered density (25–50%); category 3) heterogeneously dense (51–75%); and category 4) extremely dense (>75%). Finally, we categorized MD into two groups: “low” breast density (categories 1+2) *vs.* “high” breast density (categories 3+4).

### 2.3. Air pollution exposure assessment

#### 2.3.1. Air quality data gathering

The Madrid City Council has an Air Quality Monitoring System that comprises 24 automatic remote monitoring stations (12 urban background, 9 traffic, and 3 suburban stations) located throughout all districts. The urban background stations are representative of the exposure of the urban population in general. The traffic stations are located in areas where the pollution level is mainly influenced by emissions from a nearby street or road. The suburban stations are located on the outskirts of the city, where the highest ozone levels are found. Information on the monitoring stations is publicly available on the Madrid City Council’s Open Data website [37]. For the present paper, we selected the following eight pollutants, according to similar previous studies [25, 26]: SO_2_ (measured by ultraviolet fluorescence at 10 monitoring stations), CO (measured by infrared absorption at 9 stations), NO, NO_2_, and NO_x_ (measured by chemiluminescence at 24 stations), PM_2.5_ (measured by microbalance at 6 stations), PM_10_ (measured by microbalance at 12 stations); and O_3_ (measured by ultraviolet absorption at 14 stations). Hourly data (concentrations) of these pollutants were collected over a 3-year period (2013–2015), and annual averages were calculated. It should be noted that data not validated by the City Council were discarded for the calculation of annual averages, as well as those hourly data for which no information was available.

#### 2.3.2 Individual long-term exposure assessment

In order to ascertain the long-term residential exposure of each woman to the aforementioned pollutants, the monitoring stations were geocoded into EPSG:23030 coordinates, and ordinary kriging was applied to the 3-year annual mean concentrations of each pollutant to interpolate the surface of the municipality of Madrid. Then, each woman was assigned the interpolated concentration for each pollutant corresponding to her residence to estimate the 3-year annual mean of exposure. Finally, this exposure variable for each pollutant was categorized into quartiles based on its distribution in the study population.

Kriging is a geostatistical method of spatial interpolation of data used to estimate the value of a variable over a continuous spatial field. Ordinary kriging is one of the most widely used kriging methods and uses weighted averages of neighboring points to determine non-measured values or interpolation points. It is considered to be the best linear unbiased estimator [38–40]. Figure 1 shows the interpolated values (contour maps) for each air pollutant in the municipality of Madrid using ordinary kriging, as well as the monitoring stations.

Because air pollutants are often correlated, a correlation analysis was performed using Pearson’s correlation coefficients. The Pearson’s correlation coefficient, which varies between −1 and +1, is a statistical tool commonly used in linear regression that measures the degree of linear association between two variables, and has been used to measure the strength of the relationships among the eight pollutants analyzed in our study. Subsequently, we performed a principal component analysis (PCA), a technique of multivariate data analysis, to reduce the dimensionality of this set of eight correlated exposure variables (interpolated values for each pollutant using ordinary kriging) into a smaller set of new uncorrelated or independent variables called principal components (PCs), with a minimal loss of information [41]. These PCs are linear combinations of the original variables (air pollutants) that allow the visualization of correlations and patterns among the pollutants, thus identifying potential emission sources. PCs with eigenvalues >1 were considered in our analyses (Kaiser or latent root criterion).

### 2.4. Statistical analyses

We examined descriptive characteristics of the females by calculating absolute figures and percentages. Percent MD was calculated according to these characteristics, and we presented means along with their corresponding standard deviations and 95% confidence intervals (95%CIs). We used two-sided χ^2^ test to compare descriptive characteristics between participants with “low” and “high” breast density.

Two analyses were performed to evaluate the association between MD and residential exposure to the selected air pollutants:

1) Analysis 1 (MD as a continuous variable). The percentage of MD was log-transformed to achieve the statistical assumptions for linear regression (linearity, homoscedasticity, independence of errors, and normally distributed residuals) [42, 43]. Subsequently, the estimated β coefficients and standard errors were then exponentiated to calculate the relative change in the adjusted geometric mean of the percent MD (e^β^), comparing participants among the different exposure categories [44]. Multiple linear regression models were used to estimate e^β^ and its 95%CI for each quartile of exposure (considering Q1 as the reference), for each pollutant (8 independent models in total). Finally, regression diagnostics were checked for all the models by means of graphical analysis of residuals (standardized residuals *vs.* fitted values; means and standard deviations of the standardized residuals by deciles of fitted values; and normal Q-Q plots).
2) Analysis 2 (MD as a dichotomous variable: “high” breast density/“low” breast density). Multiple logistic regression models were used to estimate odds ratios (ORs) and 95%CIs, which represent the risk of having a higher MD in each quartile of exposure compared to Q1 as the reference quartile, for each pollutant (8 independent models, in total). The dependent variable was categorized as “low” MD = 0 and “high” MD = 1. The main assumptions used in logistic regression are: the conditional distribution of the outcome variable follows a binomial distribution, linearity in the logit (linear relationship between the natural logarithm of the odds and the continuous independent variables included in the model), independence between observations, and the binomial rather than the normal distribution describes the distribution of the errors. Logistic regression is a powerful tool, especially in epidemiologic studies, that allows multiple covariates to be analyzed simultaneously, thus reducing the effect of confounding factors [45]. Finally, the significance of each model used in the analysis was tested by means of the likelihood ratio test, calculating the difference in residuals (deviance) between the model with predictors and the model without predictors (null model).

Finally, the association between MD and residential exposure to PCs (or combined air pollutants) was assessed by applying the methodology used in the two previous analyses (for specific pollutants) to the PCs with eigenvalues >1 obtained from the PCA.

All statistical models were adjusted for recognized and potential confounding factors related to MD [22]: age and BMI (as continuous); and education, alcohol and tobacco consumption, previous breast biopsies, parity, family history of breast cancer, energy intake, and oral contraceptives use (as categorical).

For power calculations, assuming that 50% of women are exposed to high levels of air pollution, and with a sample size of 385 women for each pair of extreme quartiles between which pollution exposure is to be compared, we expected to detect a level of difference in the log-transformed percent MD in each exposure quartile of 0.09, 0.11, and 0.14 with a power of 40, 60%, and 80%, respectively. On the other hand, with a sample size of 300 participants with “low” MD and 100 with “high” MD for each pair of extreme quartiles between which pollution exposure is to be compared, we expected to detect ORs of having “high” breast density in each exposure quartile of 1.49 (or, inversely, 0.67), 1.68 (or, inversely, 0.59), and 1.94 (or, inversely, 0.52) with a power of 40, 60, and 80%, respectively.

Analyses were performed using R 4.3.0 and Stata/IC 16.1 (StataCorp LLC) software, and QGIS 3.28.16.

## 3. Results

### 3.1. Main characteristics of the study population (Table 1)

The majority of participants were younger than 45 years (52.1%), had a healthy weight (68.1%), had a university grade (64.1%), consumed <10 g/day of alcohol (67.0%), and had no previous biopsies (90.5%). MD (mean ± SD) was higher in women younger than 45 years old (36.8 ± 17.6), with a normal BMI (39.3 ± 16.8), having a university education (36.1 ± 17.9), never smokers (37.2 ± 18.6), and nulliparous (37.7 ± 18.5). Finally, the group with “low” density had a statistically significant higher number of women with obesity or overweight (BMI ≥ 25) and no previous biopsies (*p*-values < 0.001 and 0.034, respectively). Conversely, the number of nulliparous participants and the number of women who had never used oral contraceptives was higher in the group with “high” density (*p*-values = 0.005 and 0.006, respectively).

**Table 1:** Descriptive characteristics of the participants.

**Characteristics**	**All participants**			**Participants with “low” breast density**			**Participants with “high” breast density**			***p*-value^b^**
		
	**n (%)**	**Mammographic density (%)**		**n (%)**	**Mammographic density (%)**		**n (%)**	**Mammographic density (%)**		
		
		**Mean (95%CI)**	**SD^a^**		**Mean (95%CI)**	**SD^a^**		**Mean (95%CI)**	**SD^a^**	
**Total**	769 (100.0)	35.0 (33.8; 36.2)	17.4	597 (100.0)	27.9 (26.9; 28.9)	11.9	172 (100.0)	59.6 (58.3; 61.0)	8.8	
**Age**										0.061
<45	401 (52.1)	36.8 (35.1; 38.5)	17.6	300 (50.3)	28.9 (27.6; 30.2)	11.6	101 (58.7)	60.3 (58.4; 62.2)	9.8	
≥45	368 (47.9)	33.0 (31.3; 34.8)	17.0	297 (49.7)	26.9 (25.5; 28.3)	12.2	71 (41.3)	58.8 (57.1; 60.4)	7.3	
**Body mass index (kg/m^2^)**										<0.001
<18.5	15 (2.0)	38.4 (32.0; 44.8)	12.6	12 (2.0)	34.3 (28.4; 40.1)	10.3	3 (1.7)	55.1 (51.0; 59.2)	3.6	
18.5–24.9	524 (68.1)	39.3 (37.9; 40.8)	16.8	377 (63.1)	31.1 (30.0; 32.2)	10.9	147 (85.5)	60.4 (59.0; 61.9)	9.0	
25–29.9	161 (20.9)	27.0 (24.8; 29.3)	14.7	143 (24.0)	23.4 (21.6; 25.2)	11.0	18 (10.5)	55.5 (52.4; 58.7)	6.8	
≥30	69 (9.0)	20.0 (16.7; 23.3)	14.0	65 (10.9)	18.0 (15.2; 20.9)	11.8	4 (2.3)	52.3 (50.4; 54.3)	2.0	
**Education**										0.441
Primary school or less	26 (3.4)	30.8 (23.7; 38.0)	18.7	20 (3.3)	23.7 (17.2; 30.2)	14.9	6 (3.5)	54.7 (51.2; 58.3)	4.4	
Secondary school	250 (32.5)	33.3 (31.3; 35.3)	16.2	201 (33.7)	27.4 (25.8; 29.0)	11.6	49 (28.5)	57.4 (55.3; 59.5)	7.6	
University grade	493 (64.1)	36.1 (34.5; 37.7)	17.9	376 (63.0)	28.4 (27.2; 29.6)	11.9	117 (68.0)	60.8 (59.2; 62.5)	9.2	
**Alcohol consumption (g/day)**										0.647
Never	134 (17.4)	34.5 (31.5; 37.4)	17.4	104 (17.4)	27.4 (25.1; 29.7)	11.9	30 (17.4)	59.1 (56.1; 62.1)	8.3	
<10	515 (67.0)	35.2 (33.7; 36.7)	17.7	396 (66.3)	27.8 (26.6; 29.0)	11.9	119 (69.2)	59.8 (58.1; 61.5)	9.2	
≥10	120 (15.6)	34.8 (31.9; 37.8)	16.5	97 (16.3)	29.0 (26.6; 31.4)	12.1	23 (13.4)	59.5 (56.4; 62.5)	7.5	
**Tobacco consumption**										0.098
Never	297 (38.6)	37.2 (35.1; 39.3)	18.6	220 (36.9)	28.3 (26.8; 29.9)	11.4	77 (44.8)	62.6 (60.3; 64.8)	10.0	
Former smoker	262 (34.1)	33.4 (31.4; 35.3)	16.1	214 (35.8)	28.1 (26.4; 29.8)	12.4	48 (27.9)	56.8 (54.8; 58.9)	7.4	
Current smoker	210 (27.3)	33.9 (31.6; 36.2)	17.0	163 (27.3)	27.1 (25.2; 28.9)	12.2	47 (27.3)	57.7 (55.9; 59.6)	6.5	
**Previous breast biopsies**										0.034
Yes	73 (9.5)	40.4 (36.5; 44.3)	16.9	49 (8.2)	31.1 (27.9; 34.4)	11.7	24 (14.0)	59.4 (56.4; 62.3)	7.3	
No	696 (90.5)	34.4 (33.1; 35.7)	17.4	548 (91.8)	27.6 (26.6; 28.6)	11.9	148 (86.0)	59.7 (58.2; 61.1)	9.1	
**Parity**										0.005
Nulliparous	225 (29.3)	37.7 (35.3; 40.1)	18.5	157 (26.3)	28.1 (26.2; 30.0)	12.3	68 (39.5)	59.8 (57.8; 61.8)	8.5	
1	171 (22.2)	34.4 (31.7; 37.1)	18.0	134 (22.5)	27.1 (25.1; 29.0)	11.4	37 (21.5)	61.1 (57.7; 64.6)	10.8	
2	335 (43.6)	33.8 (32.0; 35.5)	16.4	273 (45.7)	28.2 (26.7; 29.6)	12.1	62 (36.1)	58.4 (56.5; 60.4)	8.0	
>2	38 (4.9)	32.6 (27.6; 37.5)	15.6	33 (5.5)	28.3 (24.4; 32.3)	11.6	5 (2.9)	60.7 (54.6; 66.8)	7.0	
**Family history of breast cancer**										0.451
None	589 (76.6)	35.2 (33.8; 36.6)	17.3	455 (76.2)	28.1 (27.0; 29.2)	12.0	134 (77.9)	59.3 (57.9; 60.8)	8.6	
Second degree only	128 (16.6)	34.7 (31.5; 37.9)	18.4	98 (16.4)	26.8 (24.4; 29.1)	12.0	30 (17.4)	60.6 (57.1; 64.1)	9.7	
First degree	52 (6.8)	33.4 (28.9; 38.0)	16.7	44 (7.4)	28.3 (24.8; 31.8)	11.8	8 (4.7)	61.6 (54.7; 68.5)	10.0	
**Energy intake (kcal/day)^c^**										0.333
>2172.5	255 (33.1)	34.9 (32.7; 37.1)	18.1	192 (32.2)	26.8 (25.1; 28.5)	12.1	63 (36.6)	59.6 (57.7; 61.6)	7.9	
1657.6–2172.5	258 (33.6)	35.9 (33.8; 37.9)	16.5	208 (34.8)	30.0 (28.4; 31.6)	11.6	50 (29.1)	60.3 (57.3; 63.3)	10.7	
<1657.6	256 (33.3)	34.2 (32.0; 36.4)	17.7	197 (33.0)	26.8 (25.1; 28.4)	12.0	59 (34.3)	59.1 (57.0; 61.1)	8.1	
**Oral contraceptives consumption**										0.006
Current use	26 (3.4)	32.3 (25.5; 39.2)	17.9	23 (3.8)	27.4 (23.1; 31.7)	10.5	3 (1.7)	70.0 (47.5; 92.4)	19.8	
Past use	423 (55.0)	33.5 (31.9; 35.1)	16.5	343 (57.5)	27.7 (26.5; 29.0)	12.2	80 (46.5)	58.1 (56.5; 59.7)	7.2	
Never	320 (41.6)	37.2 (35.2; 39.2)	18.4	231 (38.7)	28.2 (26.7; 29.7)	11.8	89 (51.8)	60.7 (58.7; 62.6)	9.4	

^a^ Standard deviation.

^b^ Two-sided χ2 test was used to compare descriptive characteristics between participants with “low” and “high” breast density.

^c^ Variable in tertiles.

### 3.2. Results of the analysis 1 (MD as a continuous variable) (Table 2)

No association was detected between MD and exposure to any of the pollutants, where the results were very close to the unity for all exposure quartiles. In the case of NO, NO_x_, and PM_10_, the results showed non-statistically significant decreased MD in all exposure quartiles, with no dose-response trends (*p*-trends = 0.646, 0.798, and 0.470, respectively).

**Table 2:** Association between log-transformed percentage of mammographic density and residential exposure to specific air pollutants.

**Pollutant exposure quartile**	**n**	**e^β^ (95%CI)^a^**	***p*-trend**
**SO_2_ (µg/m^3^)**			
Q1 (reference)	194	1	
Q2	189	1.00 (0.89–1.13)	
Q3	190	0.96 (0.85–1.08)	
Q4	196	1.05 (0.93–1.18)	0.571
**CO (mg/m^3^)**			
Q1 (reference)	189	1	
Q2	192	0.98 (0.87–1.10)	
Q3	192	1.01 (0.89–1.14)	
Q4	196	1.01 (0.89–1.14)	0.775
**NO (µg/m^3^)**			
Q1 (reference)	198	1	
Q2	185	0.90 (0.80–1.02)	
Q3	197	0.93 (0.82–1.04)	
Q4	189	0.96 (0.86–1.09)	0.646
**NO_2_ (µg/m^3^)**			
Q1 (reference)	197	1	
Q2	193	0.97 (0.86–1.09)	
Q3	190	1.03 (0.91–1.16)	
Q4	189	0.96 (0.85–1.08)	0.761
**NO_x_ (µg/m^3^)**			
Q1 (reference)	195	1	
Q2	189	0.93 (0.83–1.05)	
Q3	196	0.96 (0.85–1.07)	
Q4	189	0.98 (0.87–1.10)	0.798
**PM_2.5_ (µg/m^3^)**			
Q1 (reference)	197	1	
Q2	188	0.99 (0.88–1.11)	
Q3	188	0.98 (0.87–1.10)	
Q4	196	1.02 (0.91–1.15)	0.803
**PM_10_ (µg/m^3^)**			
Q1 (reference)	192	1	
Q2	195	0.97 (0.86–1.09)	
Q3	189	0.94 (0.83–1.06)	
Q4	193	0.96 (0.85–1.08)	0.470
**O_3_ (µg/m^3^)**			
Q1 (reference)	188	1	
Q2	198	1.04 (0.92–1.17)	
Q3	185	1.00 (0.88–1.13)	
Q4	198	1.01 (0.90–1.14)	1.00

^a^ e^β^ estimated using multiple linear regression models, adjusted for age, body mass index, education, alcohol consumption, tobacco consumption, previous breast biopsies, parity, family history of breast cancer, energy intake, and oral contraceptives consumption (an independent model for each pollutant).

### 3.3. Results of the analysis 2 (MD as a dichotomous variable: “high” density/“low” density) (Table 3)

Our results showed no association between risk of higher MD and exposure to any of the pollutants. However, non-statistically significant ORs > 1 were found in women exposed to all exposure quartiles for SO_2_, especially in the highest exposure quartile (OR_Q2_ = 1.32, OR_Q3_ = 1.10, OR_Q4_ = 1.50), and CO (OR_Q2_ = 1.14, OR_Q3_ = 1.04, OR_Q4_ = 1.05). On the other hand, O_3_ and PM_2.5_ showed ORs > 1 in the highest exposure quartile (OR_Q4_ = 1.10 and 1.27, respectively), and ORs ≤ 1 in the remaining exposure quartiles. Finally, non-statistically significant ORs < 1 were detected in all exposure quartiles for NO, NO_2_, NO_x_, and PM_10_.

**Table 3:** ORs of having “high” breast density and residential exposure to specific air pollutants.

**Pollutant exposure quartile**	**“Low” ** **breast ** **density (n)**	**“High” ** **breast ** **density (n)**	**OR (95%CI)^a^**	***p*-trend**
**SO_2_ (µg/m^3^)**				
Q1 (reference)	155	39	1	
Q2	147	42	1.32 (0.78–2.25)	
Q3	153	37	1.10 (0.64–1.89)	
Q4	142	54	1.50 (0.90–2.48)	0.197
**CO (mg/m^3^)**				
Q1 (reference)	150	39	1	
Q2	149	43	1.14 (0.67–1.92)	
Q3	147	45	1.04 (0.62–1.76)	
Q4	151	45	1.05 (0.62–1.77)	0.951
**NO (µg/m^3^)**				
Q1 (reference)	145	53	1	
Q2	148	37	0.72 (0.43–1.20)	
Q3	155	42	0.68 (0.41–1.13)	
Q4	149	40	0.78 (0.47–1.30)	0.296
**NO_2_ (µg/m^3^)**				
Q1 (reference)	150	47	1	
Q2	149	44	0.87 (0.52–1.44)	
Q3	145	45	0.98 (0.59–1.62)	
Q4	153	36	0.71 (0.42–1.21)	0.298
**NO_x_ (µg/m^3^)**				
Q1 (reference)	146	49	1	
Q2	149	40	0.77 (0.46–1.29)	
Q3	154	42	0.72 (0.43–1.20)	
Q4	148	41	0.83 (0.50–1.39)	0.432
**PM_2.5_ (µg/m^3^)**				
Q1 (reference)	153	44	1	
Q2	146	42	1.00 (0.60–1.67)	
Q3	152	36	0.86 (0.51–1.46)	
Q4	146	50	1.27 (0.77–2.10)	0.455
**PM_10_ (µg/m^3^)**				
Q1 (reference)	142	50	1	
Q2	157	38	0.69 (0.41–1.15)	
Q3	146	43	0.82 (0.49–1.36)	
Q4	152	41	0.72 (0.43–1.19)	0.303
**O_3_ (µg/m^3^)**				
Q1 (reference)	147	41	1	
Q2	155	43	0.88 (0.52–1.48)	
Q3	146	39	0.80 (0.47–1.36)	
Q4	149	49	1.10 (0.66–1.83)	0.774

^a^ ORs estimated using multiple logistic regression models, adjusted for age, body mass index, education, alcohol consumption, tobacco consumption, previous breast biopsies, parity, family history of breast cancer, energy intake, and oral contraceptives consumption (an independent model for each pollutant).

### 3.4. Results of the correlation and principal component analyses

**Fig. 2:**
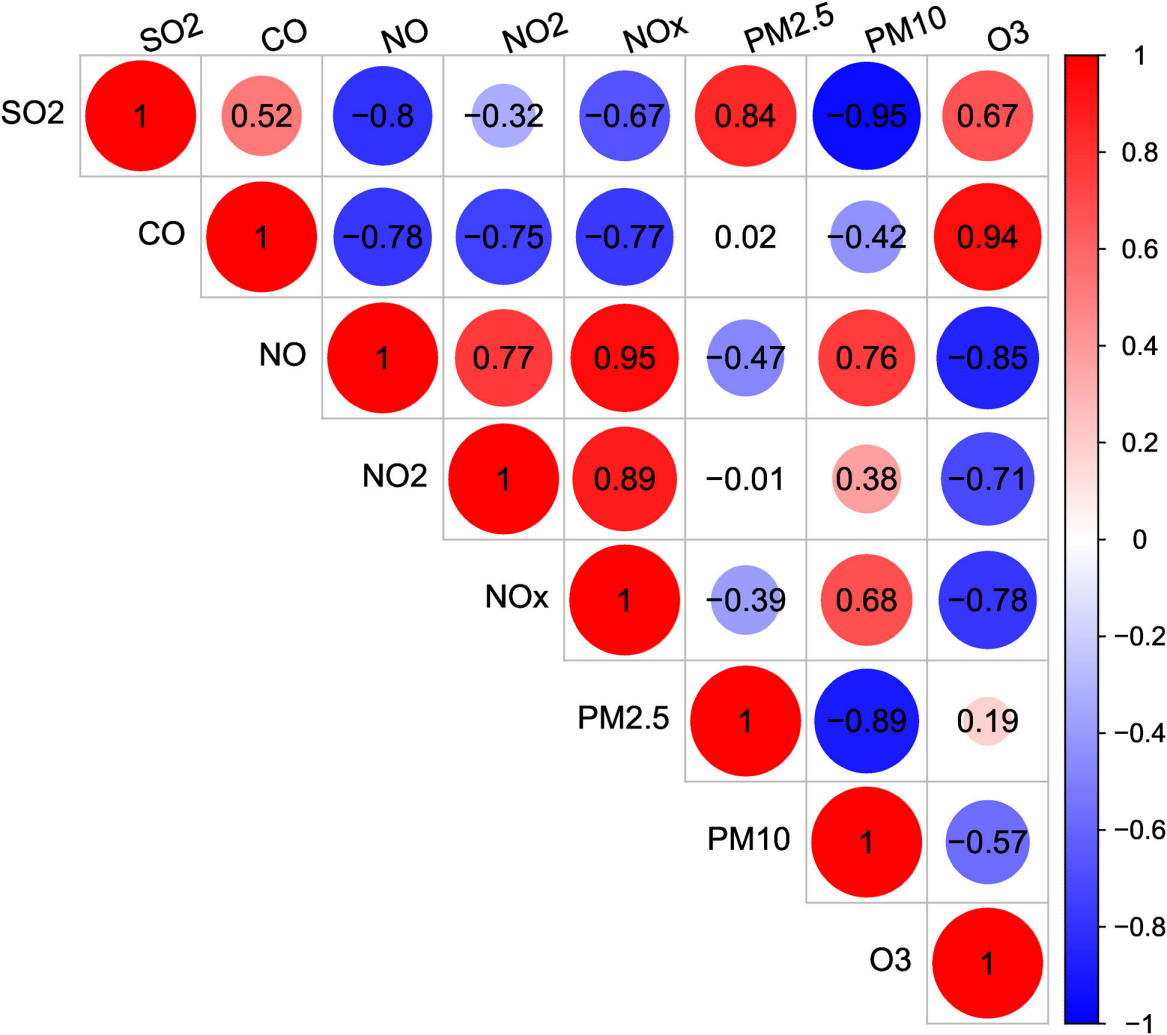
Correlation matrix among air pollutants.

Correlation analysis (Fig. 2) showed positive Pearson’s correlation coefficients >0.80 between SO_2_ and PM_2.5_ (0.84), CO and O_3_ (0.94), NO and NO_x_ (0.95), and NO_2_ and NO_x_ (0.89). On the contrary, negative Pearson’s correlation coefficients lower than −0.80 were detected between SO_2_ and PM_10_ (−0.95), NO and O_3_ (−0.85), and PM_2.5_ and PM_10_ (−0.89).

The PCA identified two PCs with eigenvalues >1 (Table 4), accounting for 92.19% of the total variance (69.35% corresponding to PC1 and 22.84% corresponding to PC2). The PCA loadings, which indicate the relative weight of each pollutant within the corresponding PC, are presented in Table 5: the first PC (PC1) shows positive loadings for NO, NO_2_, NO_x_, and PM_10_, and negative loadings for the remaining pollutants. This PC would be associated with pollutants related to traffic pollution, inasmuch as it would represent the origin of nitrogen oxides and their subsequent photochemical reaction to produce ozone from road traffic [39]. The second PC (PC2) shows positive loadings for PM_10_, CO, and O_3_, and negative loadings for the remaining pollutants. In particular, this PC discriminates between PM_10_ (loading = 0.3874) and PM_2.5_ (loading = −0.6135); this fact could suggest the influence of other sources of PM other than photochemical reactions, such as episodes of Saharan dust intrusions and/or soil abrasion [39, 46, 47]. On the other hand, one of the main sources of CO is from wildfires [48, 49] and, in this regard, during the period 2013–2015 there were 986 wildfires in the Madrid region, affecting an area of 2285 ha [50]. Finally, O_3_ is a pollutant that is not directly released by primary sources, but is formed through complex reactions in the atmosphere driven by the energy transferred to NO_2_ molecules when they absorb light from solar radiation [49]. Therefore, PC2 would be associated with “natural pollution”, i.e., pollution of natural origin that is not due to anthropogenic sources resulting from human activities.

**Table 4:** Eigenvalues, explained variance, and cumulative percentage of the principal components (PCs).

**PC number**	**Eigenvalue**	**Explained ** **variance (%)**	**Cumulative ** **percentage**
1	5.548	69.35	69.35
2	1.827	22.84	92.19
3	0.462	5.77	97.96
4	0.083	1.04	99.00
5	0.054	0.68	99.68
6	0.013	0.17	99.85
7	0.010	0.12	99.97
8	0.002	0.03	100.00

**Table 5:** PCA loadings.

**Pollutant**	**PC1**	**PC2**	**PC3**	**PC4**	**PC5**	**PC6**	**PC7**	**PC8**
SO_2_	−0.3642	−0.3492	0.2650	−0.1618	−0.0666	0.5133	−0.5131	0.3433
CO	−0.3438	0.3542	0.4333	0.1913	0.6757	0.1368	0.0616	−0.2301
NO	0.4139	−0.0489	0.0988	0.6592	0.1977	−0.2016	−0.3771	0.4000
NO_2_	0.3177	−0.3901	0.5571	−0.4090	0.2235	−0.2556	0.3220	0.2243
NO_x_	0.3973	−0.1377	0.4041	0.2533	−0.3324	0.4058	0.0109	−0.5661
PM_2.5_	−0.2311	−0.6135	−0.1453	0.0756	0.2125	−0.4044	−0.2860	−0.5030
PM_10_	0.3550	0.3874	0.0421	−0.5038	0.1056	−0.1364	−0.6272	−0.2125
O_3_	−0.3737	0.2295	0.4856	0.1082	−0.5322	−0.5148	−0.1079	0.0183

Table 6 shows the association between the log-transformed percentage of MD and proximity to PC1 (“traffic pollution”) and PC2 (“natural pollution”). No association was detected with the two PCs, where the results were very close to the unity in all exposure quartiles of PC1. In the case of PC2, the results showed non-statistically significant decreased MD in exposure quartiles Q2 and Q3 (e^β^ = 0.89 in both cases).

**Table 6:** Association between log-transformed percentage of mammographic density and residential exposure to principal components (PCs).

**PC exposure quartile**	**n**	**e^β^ (95%CI)^a^**	***p*-trend**
**PC1: “traffic pollution”**			
Q1 (reference)	198	1	
Q2	187	0.94 (0.83–1.06)	
Q3	193	0.97 (0.86–1.09)	
Q4	191	1.01 (0.89–1.13)	0.828
**PC2: “natural pollution”**			
Q1 (reference)	189	1	
Q2	190	0.89 (0.79–1.00)	
Q3	194	0.89 (0.79–1.00)	
Q4	196	0.99 (0.88–1.11)	0.850

^a^ e^β^ estimated using multiple linear regression models, adjusted for age, body mass index, education, alcohol consumption, tobacco consumption, previous breast biopsies, parity, family history of breast cancer, energy intake, and oral contraceptives consumption (an independent model for each PC).

Finally, our results showed no association between risk of having “high” MD and exposure to any of the PCs (Table 7). Non-statistically significant ORs < 1 were detected in all exposure quartiles for PC1, and in Q2 and Q3 for PC2.

**Table 7:** ORs of having “high” breast density and residential exposure to principal components (PCs).

**PC exposure ** **quartile**	**“Low” breast density (n)**	**“High” breast density (n)**	**OR (95%CI)^a^**	***p*-trend**
**PC1: “traffic pollution”**				
Q1 (reference)	149	49	1	
Q2	147	40	0.85 (0.51–1.42)	
Q3	152	41	0.81 (0.49–1.35)	
Q4	149	42	0.88 (0.53–1.46)	0.589

**PC2: “natural pollution”**				
Q1 (reference)	148	41	1	
Q2	149	41	0.91 (0.54–1.53)	
Q3	151	43	0.98 (0.58–1.63)	
Q4	149	47	1.03 (0.62–1.73)	0.835

^a^ ORs estimated using multiple logistic regression models, adjusted for age, body mass index, education, alcohol consumption, tobacco consumption, previous breast biopsies, parity, family history of breast cancer, energy intake, and oral contraceptives consumption (an independent model for each PC).

## 4. Discussion

This study examines the association between MD and exposure to eight specific ambient pollutants in premenopausal women residing in the municipality of Madrid. MD, one of the most important biomarkers of breast cancer risk, is characterized by its modifiable nature, which makes it susceptible to the influence of different exposures. This vulnerability led us to examine the possible effect of exposure to air pollutants. In general, our results revealed no association between residential exposure to air pollutants and MD in premenopausal participants. However, we detected non-statistically significant increased risks of having high MD in women exposed to the highest concentrations of SO_2_, PM_2.5_, and O_3_, and conversely, non-statistically significant decreased risks of having high MD in women exposed to NO, NO_2_, NO_x_, and PM_10_. On the other hand, PCA did not detect an association between MD and residential exposure to “traffic” and “natural” pollution.

Breast tissue changes may be influenced by several factors. Research has linked ambient pollution to an increased risk of breast tumors in urban environments [8]. On the other hand, some studies have also shown that females residing in urban zones have higher MD compared to those in rural zones [51, 52]. Furthermore, MD has been associated with ambient pollution [29, 30]. TRAP present in urban areas includes substances such as EDCs and carcinogens, which have the potential to influence the endocrine system and potentially may impact mammary gland development, ultimately leading to possible modifications in MD [53]. Lastly, it is important to mention that MD, by its dynamic nature, is influenced by relatively recent environmental exposures, meaning that MD at the time of assessment is likely to reflect exposure to pollutants during that specific time period. Therefore, it is critical to select air pollutant exposure data from the period closest to the time of data collection.

Although air quality data gathering was collected ten years ago, air pollution in urban areas is an ongoing problem. With respect to the temporal evolution in the air pollutant levels in Madrid city during the period 2013–2023, some pollutants have shown decreasing average changes in their annual average levels, such as PM_2.5_ (−6.1% between 2013 and 2023), and NO_2_ (−21.5%) [37], probably due to the traffic restrictions imposed in Madrid in recent years and the mobility restrictions during the COVID-19 pandemic [54, 55]. However, other pollutants have shown increased average changes in their annual levels over the same period, such as CO (1.3%), PM_10_ (8.8%), and O_3_ (12.4%) [37].

This is the first study conducted in Spain that analyzes the potential association between MD and residential exposure to concentrations of specific air pollutants. Our findings in premenopausal women support those obtained in the work by Eslami et al. [25], carried out in Iranian women, the only cross-sectional epidemiological study published to date on MD and exposure to concentrations of specific air pollutants, such as ours. In that study, conducted in women attending two hospitals in Tehran for screening mammography, the total number of eligible participants was 791, although only 396 were premenopausal. In contrast, our study had a significantly higher number of premenopausal women (n = 769). In terms of air quality data gathering, the Iranian study included 17 monitoring stations and six ambient air pollutants, whereas our study included more monitoring stations (n = 24) and air pollutants (n = 8). However, it is important to note that not all air pollutants were measured at each monitoring station, which is a limitation of our study. Both studies used a 3-year annual mean of exposure for each pollutant to assess individual long-term exposure. With respect to the statistical analyses, the Iranian study used multiple logistic regression models to evaluate the potential relationship between MD and exposure to the selected pollutants. The dependent variable MD was categorized into “high” density *vs.* “low” density (based on the BI-RADS classification), and the exposure variable for each pollutant was considered as continuous. In our study, we have attempted to replicate the methodology used by Eslami et al. with the same MD classification (“high” DM *vs.* “low” DM). However, we have refined the exposure measurement by categorizing the exposure variable for each pollutant into quartiles (analysis 2). In addition, it is important to note that we performed an additional analysis (analysis 1) using multiple linear regression models, which were not included in the work by Eslami et al., considering the log-transformed MD as a continuous variable. This analysis offers a more detailed explanation of the association between MD and exposure to ambient pollution. Finally, the previous two analyses were applied to the PCs (linear combinations of the original pollutants), with the purpose of detecting underlying patterns of common emission sources. The subsequent subsections provide a comparison of the findings from both studies and other studies.

### 4.1. Nitrogen oxides (NO_x_, NO_2_, and NO)

NO_x_, stemming from combustion processes in urban areas, comprises NO and NO_2_ (among others), primarily emitted by motorized traffic, making it a reliable marker for vehicle emissions. Specifically, NO_2_ is considered a dependable indicator of traffic, since over 75% of atmospheric NO_2_ in urban areas is attributed to road traffic [37]. Furthermore, research confirms that the highest levels of NO_x_ are detected in the vicinity of highways, which are characterized by higher traffic flows [56]. Only two studies have analyzed the relationship between MD and exposure to nitrogen oxides in premenopausal participants. Eslami et al. [25] found a non-statistically significant increased risk of high MD for each unit increase in NO_2_ (ppb) (OR = 1.035). In our study, we categorized NO_2_ exposure (in µg/m^3^) into quartiles, and we observed non-statistically significant ORs < 1 in all exposure quartiles. On the other hand, Huynh et al. [26] analyzed 839 premenopausal women included in the Danish Diet, Cancer and Health cohort (1993–2001) who attended mammographic screening in Copenhagen and, in consonance with our study, found a non-statistically significant inverse association between having mixed/dense MD and exposure to NO_x_ (OR = 0.92, per 20 µg/m^3^) and NO_2_ (OR = 0.77, per 10 µg/m^3^). The findings regarding the lack of association between long-term exposure to NO_x_/NO_2_ and MD in premenopausal women are consistent with those observed in some studies on breast cancer, where the authors have found no association between exposure to NO_2_ and breast cancer risk in premenopausal women [57], although inconsistent with others that have reported such an association [7, 58].

### 4.2. Ozone (O_3_)

In urban environments with elevated NO levels, O_3_ is rapidly consumed by the oxidation of NO to NO_2_. As a result, O_3_ levels tend to be lower in urban traffic zones than in less polluted environments. The latter receive, during the transport of air masses, the O_3_ generated in polluted urban and industrial areas, which is not consumed, due to the lack of local NO [59]. Thus, O_3_ can be considered as an indicator of air quality that has been hardly evaluated. Eslami et al. [25] did not find any significant relationship between O_3_ exposure and high MD (OR = 0.993), in line with that observed in our study. On the other hand, a study conducted in the US between 2001 and 2009 within a large population-based prospective cohort (the Breast Cancer Surveillance Consortium), showed statistically significant increased risks of having a mammogram classified as BI-RADS III (heterogeneously dense breasts) or IV (extremely dense breasts) in premenopausal women exposed to O_3_ for all exposure quartiles, except for women classified as BI-RADS IV in the highest quartile, where no association was found [30]. Finally, regarding breast cancer risk, although a previous study found no association with O_3_ exposure in premenopausal women [60]. It has been proposed that ozone might exhibit anticancer properties by promoting the generation of reactive oxygen species and relieving hypoxia [61].

### 4.3. Sulfur dioxide (SO_2_)

SO_2_ is used to assess ambient air quality, and its primary source is the combustion of fossil fuels in the industrial sector. Eslami et al. [25] found no statistically significant association between SO_2_ exposure and MD in premenopausal participants (OR = 0.683). However, our study suggests an increase in MD in premenopausal females exposed to higher levels of SO_2_. Regarding breast cancer, an Iranian study found no association between a higher incidence of breast tumors in premenopausal females and long-term exposure to SO_2_ [62].

### 4.4. Particulate matter (PM_10_ and PM_2.5_)

PM is comprised of a wide variety of compounds. PM_10_ and PM_2.5_ have a greater capability to enter the body through the respiratory tract [63]. With regard to MD, Eslami et al. [25] detected a 23% non-statistically significant increased risk of high MD in premenopausal females exposed to PM_2.5_, a result similar to that observed in our study (27%). They also detected a non-significant decrease in MD with PM_10_ exposure, again in line with our findings. On the other hand, Yaghjyan et al. [30] detected that premenopausal women exposed to the highest exposure quartiles of PM_2.5_ had higher odds of their mammograms being classified as BI-RADS III, although not consistently as BI-RADS IV. Finally, a nested case-control study carried out within two cohorts showed no association between residential exposure to PM (including PM_2.5_ and PM_10_) and MD [24]. In relation to breast tumors in premenopausal females and PM exposure, the findings are inconclusive [6, 60].

### 4.5. Limitations and strengths

A limitation of our work is the no monitoring of changes in MD over time, due to the cross-sectional nature of the study. Moreover, the lack of residential data during vulnerable stages of the female participants regarding breast tissue changes, such as menarche or pregnancy, which hinders our ability to identify potential associations during these stages. Additionally, participants were recruited from a single center in the municipality of Madrid, which may limit the external validity of our findings. This also implied that the variation in exposure levels of some pollutants was small, limiting the ability to detect possible associations. In this regard, it should also be noted that the statistical power of the study was rather limited, probably due to the small sample size, so that an association between exposure to air pollutants and MD in premenopausal women cannot be ruled out. However, previous studies on exposure to pollutants such as CO, SO_2_, PM_10_, NO_2_, or NO_x_ also point to a null association with MD in premenopausal women [24–26]. On the other hand, exposure assessment error is a challenge in spatial and spatiotemporal modeling. Given the impossibility of collecting individual exposure data, we have used interpolated data on exposure to individual pollutants; these data may be subject to both classical error (in which the measured value is an error-prone version of the true exposure, since random noise is added to a correctly measured exposure) and Berkson error (in which the measured value is a smoothed version of the true exposure, since part of the true exposure variability is not captured by the measurement process). A combination of these two errors could explain the lack of statistical associations [64, 65]. Additionally, although the models were adjusted for potential confounders that could modify MD, other environmental exposures may have affected the detection of potential associations between MD and air pollution exposures in their residential areas. Despite adjustment for a wide variety of potential risk factors, residual unmeasured confounders related to air pollution or MD may have affected our results (or may have interfered with the lack of significant associations). Lastly, some air pollutants, such as PM_2.5_ and CO, were measured only at a limited number of monitoring stations; therefore, the interpolation of their values by kriging may not be very accurate.

On the contrary, the main strength of our work lies in its novelty, since it is the first work conducted in Spain on residential exposure to concentrations of specific ambient pollutants and MD. On the other hand, the air pollution data were obtained from monitoring stations of the official register of Madrid city. Furthermore, a professional radiologist expert in the interpretation of mammograms, who demonstrated high internal consistency, assessed mammographic density on a continuous scale using DM-Scan, a validated computer-assisted method [66]. Finally, two methodological approaches were used in the analyses, considering MD as both a continuous and a dichotomous variable, and classifying the exposure variable for each air pollutant into quartiles to refine the measurement of exposure to air pollution. In addition, the potential correlations among pollutants were considered, and PCA was applied to detect underlying patterns of emission sources.

## 5. Conclusions

In general, our results show a lack of association between exposure to specific air pollutants and MD in premenopausal females in the municipality of Madrid. Further research is necessary to refute/confirm these findings, since MD is a modifiable biomarker for breast tumors, and knowing the relationship between them and the environmental factors that could modify it is fundamental to design prevention strategies.

## Abbreviations

EDCs: Endocrine-disrupting chemicals
TRAP: Traffic-related air pollution
NO_x_: Nitrogen oxides
PM: Particulate matter
NO_2_: Nitrogen dioxide
SO_2_: Sulfur dioxide
MD: Mammographic density
BMI: Body mass index
O_3_: Ozone
CO: carbon monoxide
NO: Nitric oxide/nitrogen monoxide
PM_2.5_: Particulate matter <2.5 µm
PM_10_: Particulate matter <10 µm
BI-RADS: Breast Imaging-Reporting and Data System
PCA: Principal component analysis
PCs: Principal components
95%CIs: 95% confidence intervals
ORs: Odds ratios
ISCIII: Carlos III Institute of Health

## References

[1] Bray F, Laversanne M, Sung H, Ferlay J, Siegel RL, Soerjomataram I, . Global cancer statistics 2022: GLOBOCAN estimates of incidence and mortality worldwide for 36 cancers in 185 countries. CA Cancer J Clin. 2024. doi: 10.3322/caac.21834. 38572751

[2] Ferlay J, Laversanne M, Ervik M, Lam F, Colombet M, Mery L, et al. Global Cancer Observatory: Cancer Tomorrow (version 1.1). Lyon, France: International Agency for Research on Cancer. 2024. https://gco.iarc.fr/tomorrow/en/dataviz/trends?multiple_populations=0&mode=cancer&multiple_cancers=1&types=0&sexes=2&cancers=20&populations=724 (accessed September 20, 2024).

[3] Loomis D, Grosse Y, Lauby-Secretan B, Ghissassi FE, Bouvard V, Benbrahim-Tallaa L, . The carcinogenicity of outdoor air pollution. Lancet Oncol. 2013;14:1262–3. doi: 10.1016/S1470-2045(13)70487-X. 25035875

[4] Health Effects Institute. Systematic Review and Meta-analysis of Selected Health Effects of Long-Term Exposure to Traffic-Related Air Pollution 2023. https://www.healtheffects.org/system/files/hei-special-report-23_6.pdf (accessed September 20, 2024).

[5] Liao H, Murithi RG, Lu C, Yang W, Liu Z, Cao L. Long-term exposure to traffic-related air pollution and temperature increases gynecological cancers. Build Environ. 2023;230:109989. doi: 10.1016/j.buildenv.2023.109989.

[6] Andersen ZJ, Ravnskjær L, Andersen KK, Loft S, Brandt J, Becker T, . Long-term Exposure to Fine Particulate Matter and Breast Cancer Incidence in the Danish Nurse Cohort Study. Cancer Epidemiol Biomark Prev Publ Am Assoc Cancer Res Cosponsored Am Soc Prev Oncol. 2017;26:428–30. doi: 10.1158/1055-9965.EPI-16-0578. 27913396

[7] Gabet S, Lemarchand C, Guénel P, Slama R. Breast Cancer Risk in Association with Atmospheric Pollution Exposure: A Meta-Analysis of Effect Estimates Followed by a Health Impact Assessment. Environ Health Perspect. 2021;129:057012. doi: 10.1289/EHP8419. 34038220 PMC8153692

[8] Stults WP, Wei Y. Ambient air emissions of polycyclic aromatic hydrocarbons and female breast cancer incidence in US. Med Oncol. 2018;35:88. doi: 10.1007/s12032-018-1150-3. 29730800

[9] Crouse DL, Goldberg MS, Ross NA, Chen H, Labrèche F. Postmenopausal Breast Cancer Is Associated with Exposure to Traffic-Related Air Pollution in Montreal, Canada: A Case–Control Study. Environ Health Perspect. 2010;118:1578–83. doi: 10.1289/ehp.1002221. 20923746 PMC2974696

[10] Lemarchand C, Gabet S, Cénée S, Tvardik N, Slama R, Guénel P. Breast cancer risk in relation to ambient concentrations of nitrogen dioxide and particulate matter: results of a population-based case-control study corrected for potential selection bias (the CECILE study). Environ Int. 2021;155:106604. doi: 10.1016/j.envint.2021.106604. 34030067

[11] Cheng I, Tseng C, Wu J, Yang J, Conroy SM, Shariff-Marco S, . Association between ambient air pollution and breast cancer risk: The multiethnic cohort study. Int J Cancer. 2020;146:699–711. doi: 10.1002/ijc.32308. 30924138 PMC6765455

[12] Wei Y, Davis J, Bina WF. Ambient air pollution is associated with the increased incidence of breast cancer in US. Int J Environ Health Res. 2012;22:12–21. doi: 10.1080/09603123.2011.588321. 21644128

[13] Gorham ED, Garland CF, Garland FC. Acid haze air pollution and breast and colon cancer mortality in 20 Canadian cities. Can J Public Health Rev Can Sante Publique. 1989;80:96–100. 2720547

[14] Baccarelli A, Wright RO, Bollati V, Tarantini L, Litonjua AA, Suh HH, . Rapid DNA Methylation Changes after Exposure to Traffic Particles. Am J Respir Crit Care Med. 2009;179:572–8. doi: 10.1164/rccm.200807-1097OC. 19136372 PMC2720123

[15] Madrigano J, Baccarelli A, Mittleman MA, Wright RO, Sparrow D, Vokonas PS, . Prolonged Exposure to Particulate Pollution, Genes Associated with Glutathione Pathways, and DNA Methylation in a Cohort of Older Men. Environ Health Perspect. 2011;119:977–82. doi: 10.1289/ehp.1002773. 21385671 PMC3222977

[16] McCracken J, Baccarelli A, Hoxha M, Dioni L, Melly S, Coull B, . Annual Ambient Black Carbon Associated with Shorter Telomeres in Elderly Men: Veterans Affairs Normative Aging Study. Environ Health Perspect. 2010;118:1564–70. doi: 10.1289/ehp.0901831. 21465749 PMC2974694

[17] Salam MT, Byun HM, Lurmann F, Breton CV, Wang X, Eckel SP, . Genetic and epigenetic variations in inducible nitric oxide synthase promoter, particulate pollution, and exhaled nitric oxide levels in children. J Allergy Clin Immunol. 2012;129:232–9.e7. doi: 10.1016/j.jaci.2011.09.037. 22055874 PMC3487398

[18] Vattanasit U, Navasumrit P, Khadka MB, Kanitwithayanun J, Promvijit J, Autrup H, . Oxidative DNA damage and inflammatory responses in cultured human cells and in humans exposed to traffic-related particles. Int J Hyg Environ Health. 2014;217:23–33. doi: 10.1016/j.ijheh.2013.03.002. 23567252

[19] Yaghjyan L, Arao R, Brokamp C, O’Meara ES, Sprague BL, Ghita G, . Association between air pollution and mammographic breast density in the Breast Cancer Surveilance Consortium. Breast Cancer Res. 2017;19:36. doi: 10.1186/s13058-017-0828-3. 28381271 PMC5382391

[20] White AJ, Keller JP, Zhao S, Carroll R, Kaufman JD, Sandler DP. Air Pollution, Clustering of Particulate Matter Components, and Breast Cancer in the Sister Study: A U.S.-Wide Cohort. Environ Health Perspect. 2019;127:107002. doi: 10.1289/EHP5131. 31596602 PMC6867190

[21] Boyd NF, Guo H, Martin LJ, Sun L, Stone J, Fishell E, . Mammographic density and the risk and detection of breast cancer. N Engl J Med. 2007;356:227–36. doi: 10.1056/NEJMoa062790. 17229950

[22] Huo CW, Chew GL, Britt KL, Ingman WV, Henderson MA, Hopper JL, . Mammographic density—a review on the current understanding of its association with breast cancer. Breast Cancer Res Treat. 2014;144:479–502. doi: 10.1007/s10549-014-2901-2. 24615497

[23] Bond-Smith D, Stone J. Methodological Challenges and Updated Findings from a Meta-analysis of the Association between Mammographic Density and Breast Cancer. Cancer Epidemiol Biomarkers Prev. 2019;28:22–31. doi: 10.1158/1055-9965.EPI-17-1175. 30206060

[24] DuPre NC, Hart JE, Bertrand KA, Kraft P, Laden F, Tamimi RM. Residential particulate matter and distance to roadways in relation to mammographic density: results from the Nurses’ Health Studies. Breast Cancer Res BCR. 2017;19:124. doi: 10.1186/s13058-017-0915-5. 29169389 PMC5701365

[25] Eslami B, Alipour S, Omranipour R, Naddafi K, Naghizadeh MM, Shamsipour M, . Air pollution exposure and mammographic breast density in Tehran, Iran: a cross-sectional study. Environ Health Prev Med. 2022;27:28. doi: 10.1265/ehpm.22-00027. 35786683 PMC9283909

[26] Huynh S, von Euler-Chelpin M, Raaschou-Nielsen O, Hertel O, Tjønneland A, Lynge E, . Long-term exposure to air pollution and mammographic density in the Danish Diet, Cancer and Health cohort. Environ Health Glob Access Sci Source. 2015;14:31. doi: 10.1186/s12940-015-0017-8. PMC4392475 25879829

[27] Jiménez T, Pollán M, Domínguez-Castillo A, Lucas P, Sierra MÁ, Fernández de Larrea-Baz N, . Residential proximity to industrial pollution and mammographic density. Sci Total Environ. 2022;829:154578. doi: 10.1016/j.scitotenv.2022.154578. 35304152

[28] Kotake R, Yamauchi H, Kimura T, Tsunoda H, Lee M. An association between mammographic breast density and fine particulate matter among postmenopausal women. Environ Sci Pollut Res Int. 2022;30:25953–8. doi: 10.1007/s11356-022-23529-0. 36348241

[29] White AJ, Weinberg CR, O’Meara ES, Sandler DP, Sprague BL. Airborne metals and polycyclic aromatic hydrocarbons in relation to mammographic breast density. Breast Cancer Res. 2019;21:24. doi: 10.1186/s13058-019-1110-7. 30760301 PMC6373138

[30] Yaghjyan L, Arao R, Brokamp C, O’Meara ES, Sprague BL, Ghita G, . Association between air pollution and mammographic breast density in the Breast Cancer Surveilance Consortium. Breast Cancer Res. 2017;19:36. doi: 10.1186/s13058-017-0828-3. 28381271 PMC5382391

[31] Assi V, Warwick J, Cuzick J, Duffy SW. Clinical and epidemiological issues in mammographic density. Nat Rev Clin Oncol. 2012;9:33–40. doi: 10.1038/nrclinonc.2011.173. 22143145

[32] Vilmun BM, Vejborg I, Lynge E, Lillholm M, Nielsen M, Nielsen MB, . Impact of adding breast density to breast cancer risk models: A systematic review. Eur J Radiol. 2020;127:109019. doi: 10.1016/j.ejrad.2020.109019. 32361308

[33] Lope V, Toribio MJ, Pérez-Gómez B, Castelló A, Mena-Bravo A, Sierra MÁ, . Serum 25-hydroxyvitamin D and mammographic density in premenopausal Spanish women. J Steroid Biochem Mol Biol. 2019;189:101–7. doi: 10.1016/j.jsbmb.2019.03.004. 30836177

[34] European Environmental Agency. Europe’s urban air quality. Re-assessing implementation challenges in cities. EEA Report No 24/2018. 2019. https://op.europa.eu/en/publication-detail/-/publication/158854fd-714c-11e9-9f05-01aa75ed71a1/language-en# (accessed September 20, 2024).

[35] Pollán M, Llobet R, Miranda-García J, Antón J, Casals M, Martínez I, . Validation of DM-Scan, a computer-assisted tool to assess mammographic density in full-field digital mammograms. SpringerPlus. 2013;2:242. doi: 10.1186/2193-1801-2-242. 23865000 PMC3693435

[36] D’Orsi C, Sickles E, Mendelson E. ACR BI-RADS^®^ atlas, breast imaging reporting and data system. American College of Radiology; 2003.

[37] Madrid City Council. Air Quality Annual Report 2023. General Directorate of Sustainability and Environmental Control. 2023. https://airedemadrid.madrid.es/UnidadesDescentralizadas/Sostenibilidad/CalidadAire/Publicaciones/Memorias_anuales/Ficheros/MEMORIA_2023_01.pdf (accessed September 6, 2024).

[38] Kumar A, Mishra RK, Sarma K. Mapping spatial distribution of traffic induced criteria pollutants and associated health risks using kriging interpolation tool in Delhi. J Transp Health. 2020;18:100879. doi: 10.1016/j.jth.2020.100879.

[39] Núñez-Alonso D, Pérez-Arribas LV, Manzoor S, Cáceres JO. Statistical Tools for Air Pollution Assessment: Multivariate and Spatial Analysis Studies in the Madrid Region. J Anal Methods Chem. 2019;2019:9753927. doi: 10.1155/2019/9753927. 30881728 PMC6387705

[40] Wackernagel H. Ordinary Kriging. Multivar. Geostat., Berlin, Heidelberg: Springer Berlin Heidelberg; 1995, p. 74–81. doi: 10.1007/978-3-662-03098-1_11.

[41] Hair JF Jr, Black WC, Babin BJ, Anderson RE. Multivariate data analysis. 8th ed. Andover, Hampshire (UK): Cengage Learning EMEA; 2019.

[42] Blair LK, Warner ET, James P, Hart JE, VoPham T, Barnard ME, . Exposure to natural vegetation in relation to mammographic density in a Massachusetts-based clinical cohort. Environ Epidemiol Phila Pa. 2022;6:e216. doi: 10.1097/EE9.0000000000000216. PMC9374192 35975164

[43] Jiménez T, Domínguez-Castillo A, Fernández de Larrea-Baz N, Lucas P, Sierra MÁ, Salas-Trejo D, . Residential exposure to traffic pollution and mammographic density in premenopausal women. Sci Total Environ. 2024;928:172463. doi: 10.1016/j.scitotenv.2024.172463. 38615764

[44] García-Pérez J, Pollán M, Pérez-Gómez B, González-Sánchez M, Cortés Barragán RA, Maqueda Blasco J, . Occupation and mammographic density: A population-based study (DDM-Occup). Environ Res. 2017;159:355–61. doi: 10.1016/j.envres.2017.08.028. 28843166

[45] Hosmer DW, Lemeshow S, Sturdivant RX. Applied Logistic Regression. 3rd edition. New Jersey: Wiley; 2013.

[46] Reyes M, Díaz J, Tobias A, Montero JC, Linares C. Impact of Saharan dust particles on hospital admissions in Madrid (Spain). Int J Environ Health Res. 2014;24:63–72. doi: 10.1080/09603123.2013.782604. 23544440

[47] Díaz J, Linares C, Carmona R, Russo A, Ortiz C, Salvador P, . Saharan dust intrusions in Spain: Health impacts and associated synoptic conditions. Environ Res. 2017;156:455–67. doi: 10.1016/j.envres.2017.03.047. 28412538

[48] Byrne B, Liu J, Bowman KW, Pascolini-Campbell M, Chatterjee A, Pandey S, . Carbon emissions from the 2023 Canadian wildfires. Nature. 2024. doi: 10.1038/s41586-024-07878-z. PMC11424480 39198654

[49] World Health Organization. WHO global air quality guidelines. Particulate matter (PM2.5 and PM10), ozone, nitrogen dioxide, sulfur dioxide and carbon monoxide 2021. https://iris.who.int/bitstream/handle/10665/345329/9789240034228-eng.pdf?sequence=1 (accessed September 20, 2024). 34662007

[50] Institute of Statistics of the Community of Madrid. Wildfires. 2013–2023 2024. https://www.madrid.org/iestadis/fijas/estructu/sociales/descarga/incendios.xlsx (accessed September 17, 2024).

[51] Emaus MJ, Bakker MF, Beelen RMJ, Veldhuis WB, Peeters PHM, van Gils CH. Degree of urbanization and mammographic density in Dutch breast cancer screening participants: results from the EPIC-NL cohort. Breast Cancer Res Treat. 2014;148:655–63. doi: 10.1007/s10549-014-3205-2. 25399231

[52] Perry N, Moss S, Dixon S, Milner S, Mokbel K, Lemech C, . Mammographic Breast Density and Urbanization: Interactions with BMI, Environmental, Lifestyle, and Other Patient Factors. Diagn Basel Switz. 2020;10:418. doi: 10.3390/diagnostics10060418. PMC7344692 32575725

[53] Gray JM, Rasanayagam S, Engel C, Rizzo J. State of the evidence 2017: an update on the connection between breast cancer and the environment. Environ Health. 2017;16:94. doi: 10.1186/s12940-017-0287-4. 28865460 PMC5581466

[54] Galán-Madruga D. Urban air quality changes resulting from the lockdown period due to the COVID-19 pandemic. Int J Environ Sci Technol IJEST. 2023;20:7083–98. doi: 10.1007/s13762-022-04464-6. PMC9391654 36035638

[55] Izquierdo R, García Dos Santos S, Borge R, Paz D, Sarigiannis D, Gotti A, . Health impact assessment by the implementation of Madrid City air-quality plan in 2020. Environ Res. 2020;183:109021. doi: 10.1016/j.envres.2019.109021. 32044574

[56] Spanish Ministry for Ecological Transition and the Demographic Challenge. Óxidos de Nitrógeno (nitrogen oxides) 2024. https://www.miteco.gob.es/en/calidad-y-evaluacion-ambiental/temas/atmosfera-y-calidad-del-aire/calidad-del-aire/salud/oxidos-nitrogeno.html (accessed September 20, 2024).

[57] Amadou A, Praud D, Coudon T, Deygas F, Grassot L, Dubuis M, . Long-term exposure to nitrogen dioxide air pollution and breast cancer risk: A nested case-control within the French E3N cohort study. Environ Pollut. 2023;317:120719. doi: 10.1016/j.envpol.2022.120719. 36435283

[58] Hystad P, Villeneuve PJ, Goldberg MS, Crouse DL, Johnson K. Exposure to traffic-related air pollution and the risk of developing breast cancer among women in eight Canadian provinces: A case–control study. Environ Int. 2015;74:240–8. doi: 10.1016/j.envint.2014.09.004. 25454241

[59] Spanish Ministry for Ecological Transition and the Demographic Challenge. Ozono (ozone) 2024. https://www.miteco.gob.es/en/calidad-y-evaluacion-ambiental/temas/atmosfera-y-calidad-del-aire/calidad-del-aire/salud/ozono.html (accessed September 20, 2024).

[60] White AJ, Gregoire AM, Niehoff NM, Bertrand KA, Palmer JR, Coogan PF, . Air pollution and breast cancer risk in the Black Women’s Health Study. Environ Res. 2021;194:110651. doi: 10.1016/j.envres.2020.110651. 33387538 PMC7946730

[61] Li Y, Pu R. Ozone Therapy for Breast Cancer: An Integrative Literature Review. Integr Cancer Ther. 2024;23:15347354241226668. doi: 10.1177/15347354241226667. PMC10807353 38258533

[62] Khorrami Z, Pourkhosravani M, Karamoozian A, Jafari-Khounigh A, Akbari ME, Rezapour M, . Ambient air pollutants and breast cancer stage in Tehran, Iran. Sci Rep. 2024;14:3873. doi: 10.1038/s41598-024-53038-8. 38365800 PMC10873290

[63] Spanish Ministry for Ecological Transition and the Demographic Challenge. Partículas en suspensión (particulate matter) 2024. https://www.miteco.gob.es/en/calidad-y-evaluacion-ambiental/temas/atmosfera-y-calidad-del-aire/emisiones/prob-amb/particulas.html (accessed September 20, 2024).

[64] Keller JP, Chang HH, Strickland MJ, Szpiro AA. Measurement Error Correction for Predicted Spatiotemporal Air Pollution Exposures. Epidemiology. 2017;28:338–45. doi: 10.1097/EDE.0000000000000623. 28099267 PMC5378630

[65] Szpiro AA, Paciorek CJ. Measurement error in two-stage analyses, with application to air pollution epidemiology. Environmetrics. 2013;24:501–17. doi: 10.1002/env.2233. 24764691 PMC3994141

[66] Pollán M, Llobet R, Miranda-García J, Antón J, Casals M, Martínez I, . Validation of DM-Scan, a computer-assisted tool to assess mammographic density in full-field digital mammograms. SpringerPlus. 2013;2:242. doi: 10.1186/2193-1801-2-242. 23865000 PMC3693435

